# The verification of ethnographic data

**DOI:** 10.1177/1466138117723936

**Published:** 2017-07-28

**Authors:** Robert Pool

**Affiliations:** University of Amsterdam, the Netherlands

**Keywords:** ethnography, verification, anthropology, research, data storage

## Abstract

Anthropologists are increasingly required to account for the data on which they base their interpretations and to make it available for public scrutiny and re-analysis. While this may seem straightforward (why not place our data in online repositories?), it is not. Ethnographic ‘data’ may consist of everything from verbatim transcripts (‘hard data’) to memories and impressions (‘soft data’). Hard data can be archived and re-analysed; soft data cannot. The focus on hard ‘objective’ data contributes to the delegitimizing of the soft data that are essential for ethnographic understanding, and without which hard data cannot be properly interpreted. However, the credibility of ethnographic interpretation requires the possibility of verification. This could be achieved by obligatory, standardised forms of personal storage with the option for audit if required, and by being more explicit in publications about the nature and status of the data and the process of interpretation.

Thirty years ago, James Clifford and others showed how anthropologists construct their authority in ethnographic texts by presenting evidence of their presence in the field (e.g. photos or anecdotes), and by using literary devices such as free indirect discourse to suggest that their interpretations are shared by their respondents. Apart from this critique of the textual self-verification of their authority, anthropologists have generally not been called to account for the nature and status of the data on which they base their interpretations. This is surprising, given that the lone ethnographer conducting research in a distant location, often among people unlikely to read the final ethnographic text, probably gives more scope for embellishment, or even outright fraud, than any other type of research. This is changing, however, and the growing culture of accountability and audit in academic research is beginning to impinge on ethnographic research.

In general, the reasons for wanting to make scientific research data accessible and verifiable are laudable. Making data available enables others to carry out further analysis without having to waste resources collecting new data, and it enables others to check the veracity of results, thus safeguarding against fraudulent research and the manipulation of results, especially relevant when significant political or financial interests may be involved in the outcome. Accessibility and verification contribute to the credibility of science and research more generally in a context of increasing public scepticism. Making research data accessible would also appear to be relatively straightforward. Many kinds of data are already routinely collected and stored in appropriate archives or repositories: population survey data, epidemiological data, clinical data, experimental data.

So should ethnographic data be stored and made accessible for verification purposes? Before answering this question, we must first determine what ethnographic data *is*, and what we mean – or might want to mean – by its verification.

First, what is ethnographic data? Anthropologists (and increasingly other disciplines) carry out long-term ethnographic research involving participant observation. How individual researchers do ethnography varies. At the more informal end of the continuum there are ethnographers who do not use any formal techniques to collect data – indeed, some are averse to the whole notion of ‘data’ that can be ‘collected’. These ethnographers participate in local activities, or just hang around in the community and observe, listen, ask occasional questions, and perhaps make notes. Sometimes this is a question of personal style and sometimes it is born of necessity because the setting or the topic are such that more formal methods such as interviews, or even open note taking, are not possible. At the other end of the continuum are the ethnographers who collect most or all of their data through more formal techniques that enable the direct recording of information, such as interviews, focus groups, questionnaires, photography, or pile sorts.

Ethnographic data may consist of everything from verbatim transcripts of interviews, and other physical artefacts such as photographs or lists, to the memories and impressions of the ethnographer. All ethnographers come back from the field with a store of this more ‘subjective’ and partly subconscious information. Some consider it their prime data, while for others it is extraneous and not considered data. I have had more than one PhD student with dull and uninteresting verbatim transcriptions of interviews who was unwilling to use the rich and relevant information stored in his head because it was not ‘data’, and I once co-supervised a student whose supervisor forbade her to use any information in her thesis that was not derived from her interview transcripts for the same reason.

Even if it were desirable to exclude such informal information from the interpretation, it would not be feasible because the very experience of immersion during extensive fieldwork necessarily colours any interpretation of the more formal data. I am talking here not only of the role of information gathered during informal conversations, or casually overheard or observed, but also of the transformative nature of fieldwork itself: the ethnographer who emerges after such fieldwork is sometimes no longer quite the same person who embarked on it. In other words, part of the ‘data’ is embodied in the researcher.

So ethnographic data cover a broad ontological range from ‘hard, objective’ documents and other physical artefacts to ‘soft, subjective’ memories and experiences. And the problem is not so much that it is difficult to draw a clear line between the two, but that ethnographic data are hybrid: the hardest verbatim transcripts are subject to continual reinterpretation based on the memory and experience of the researcher, and the softest memories and impressions can easily be hardened into texts such as field reports. Soft, subjective information is already buried in the hard, objective data through its very collection.

Second, what would accessibility and verification mean, practically, in relation to such hybrid hard-soft data? It would mean that only physical artefact data, such as documents and recordings, would be stored and verified. For this to work these artefacts would need to be legible (not everyone’s field notes are legible), and there would need to be some level of interpretability (a photo of a mountain would be meaningless unless it was clear that this was a particular mountain in Cameroon where witches were said to gather, for example). Consequently, ethnographic data repositories would end up containing only certain types of legible, physical data, and those would be the only data that would be amenable to verification. As a result, ethnographers whose data are mainly soft would be exposed to less scrutiny than those with hard artefact data.

At the same time, insisting on the availability of physical data as a verification of research carried out would contribute to delegitimizing the soft data that are essential for ethnographic understanding. This tendency is already marked in academic publishing, where journal reviewers and editors increasingly demand that qualitative researchers present their ‘data’ in the form of quotes (as though such fragments are the qualitative equivalent of a statistician’s numbers), and expect methodological justification for the selection of quotes through the use of coding software. In many journals, soft data are rejected as hearsay and anecdote.

Making data accessible for verification would not necessarily prevent fraud, as dishonest ethnographers who invent and embellish data in their publications could just as easily invent and embellish their basic data as well, before making it available. I once recruited a local graduate to carry out parallel research to mine in a neighbouring village in Cameroon. I explained my hypothesis and he worked hard and delivered many detailed and fascinating interview transcripts which confirmed exactly what I had hypothesized. The problem was that my own interviews revealed that I had been mistaken. I spoke to his assistant, who confessed that he, the graduate, had never left the house and had invented everything. The graduate was sly and creative, and if it were not for the fact that I got it wrong to start with and was in the next village, I might never have known.

But the problem of verification runs deeper than that. In an ethnographic study of euthanasia decision-making in a hospital, my respondents found some of their statements in my verbatim transcripts shocking, not because they hadn’t made them, but because they had. They found that in print, on the page, the statements seemed a lot harsher than they remembered or had intended. I experienced a similar clash during transcription: what I was typing from the tape seemed much harsher than my memory of those same statements in the context of the actual interview.

This raises the question of whether verbatim is the most accurate way to transcribe (and what ‘accuracy’ should mean in this context). It also has consequences for archiving and verification, and for any attempt to build new research on existing data. What if the archived texts do not represent what ‘really’ transpired because their interpretation (and thus their verification) requires that they be made properly legible through the multiple perspectives of the participants – i.e. it requires the soft data that, by its very nature, cannot be archived?

This is further complicated by potential ambiguity relating to the genre of the speech act from which data are collected. When Charles Briggs reflected on misunderstandings during his interviews with a couple of potters to whom he was apprenticed as part of his dissertation fieldwork, he realized that this was because he had assumed that the speech acts he was recording were interviews (he was the expert interviewing them), whereas the potters saw them as instruction sessions (they were the experts teaching him). If a third party attempted to verify conversations stored and classified as interviews with respondents who interpreted them as training sessions, without taking both parties’ soft data into account, then the conclusion might well be that the data failed the verification test.

All this seems to point to the futility of any attempt to verify ethnographic data. Not so. Ethnographers – perhaps more than other researchers – need to be held to some kind of accountability, particularly *because* of the nature of their data. And the impossibility of comprehensive accessibility and verification does not necessarily mean that there should be no attempt at all.

I once hired anthropological consultants to do some research for me. In addition to a report, the deliverables included the completed survey questionnaires and the transcribed open interviews and field notes on which the report was based. The report, when they delivered it, looked excellent and ready to publish: it was detailed, and full of quotes and tables. I asked for the other deliverables. The anthropologists were stunned: what did I want those for; everything was in the report. Finally, after threats of legal action, they sent the data. The tables did not tally with the questionnaires and the longest transcript of the ‘open in-depth interviews’ was a short paragraph. What did I expect, they asked: this was ethnography and the data were in their heads. Don’t tell me *you* have piles of interviews and notes from your own fieldwork, one of them said, incredulously, because I don’t know any anthropologist who does.

The thing is, *I do*. And those piles of documents, photos, and tapes, together with the more recent electronic files, would make a substantial difference in the credibility of my ethnographic research, were anyone to question it, in spite of all the caveats pertaining to such stored data discussed above. That is because any third party would, should I grant them access, at the very least be able to verify that I had actually been there and interviewed the people I claim to have interviewed.

So is some sort of public repository the way to go after all? I don’t think so, for the reasons discussed above. Hard ethnographic data are partial and personal, making simple checking of the basic ‘facts’ feasible, but any re-use or re-interpretation of findings difficult in the absence of the soft component. I would be reluctant to make available in this way my informants’ sometimes personal and intimate stories because that is not what they would have had in mind when they entrusted them to me – and it is not what I had in mind when I elicited them and offered them my own stories in exchange. Moreover, the prospect of public storage, verification and potential re-use outside the control of the researchers and their respondents would affect the way in which data are collected and recorded, as well as respondents’ willingness to discuss sensitive or dangerous issues and ethnographers’ willingness to ask about them.

I have three suggestions. First, storage in repositories is not necessary for basic fact-checking. Ethnographers should continue (or start if they do not already do so) to store their physical data in dusty boxes in garage or attic or, for contemporary research, on encrypted digital backup drives. But make the practice part of the disciplinary code of ethical conduct and accountability, and develop a standard set of guidelines – disciplinary best practice – on how to do this. Simple recording of dates and locations of interviews, details of respondents (where feasible), etc., seem pretty basic and reasonable to me. One can easily imagine situations in which this is not a good idea because it might endanger respondents or researcher, but these situations are not all that common and the reasons for not recording such data can easily be noted and archived.

Also include in that code a clear rejection of the requirement by some ethics committees that qualitative researchers destroy recordings after transcription and then destroy the transcriptions after analysis in the name of confidentiality – an unethical requirement if such destruction is not what study participants agreed to or expected when they told their stories, and because it prevents the very verification that such committees should be defending. Peers, professional organizations, and ethics committees – acceptable to researcher and, ideally, respondents as well – would be potential third parties who could carry out verification should it be required.

Second, ethnographers can already go a long way toward increasing the accessibility, verifiability and legitimacy of their data in their publications. Although norms are changing somewhat, it is still common to find ethnographies, articles based on ethnographic research and anthropology PhD dissertations that give no indication whatever of the duration of fieldwork or the number of people spoken to, and no description of the methods used to collect data. Silence on this (and on the ethnographer’s proficiency in the local vernacular) does not instil confidence in the data and the interpretations.

Third, ethnographers could present more of their data in their ethnographies – i.e. allow their participants more say. Ethnographers tend to be parsimonious in their presentation of the voices of their interlocutors – free indirect discourse still reigns. Rather than confining their voices to the box in the attic or the backup drive, why not give them more space in the final ethnographic text? I am not talking about the token quotes so loved by reviewers and editors of some journals, but substantial passages of discourse. In that way, more of the data are already out there and open to at least some sort of verification.

